# RANKL-mediated harmonious dialogue between fetus and mother guarantees smooth gestation by inducing decidual M2 macrophage polarization

**DOI:** 10.1038/cddis.2017.505

**Published:** 2017-10-12

**Authors:** Yu-Han Meng, Wen-Jie Zhou, Li-Ping Jin, Li-Bing Liu, Kai-Kai Chang, Jie Mei, Hui Li, Jian Wang, Da-Jin Li, Ming-Qing Li

**Affiliations:** 1 Laboratory for Reproductive Immunology, Hospital of Obstetrics and Gynecology, Fudan University Shanghai Medical College, Shanghai, People’s Republic of China; 2 Key Laboratory of Reproduction Regulation of NPFPC, SIPPR, IRD, Fudan University, Shanghai, People’s Republic of China; 3 Shanghai Key Laboratory of Female Reproductive Endocrine Related Diseases, Hospital of Obstetrics and Gynecology, Fudan University Shanghai Medical College, Shanghai, People’s Republic of China; 4 Clinical and Translational Research Center, Shanghai First Maternity and Infant Hospital, Tongji University School of Medicine, Shanghai, People’s Republic of China

## Abstract

Decidual macrophages (dM*φ*) contribute to maternal–fetal tolerance. However, the mechanism of dM*φ* differentiation during pregnancy is still largely unknown. Here, we report that receptor activator for nuclear factor-*κ* B ligand (RANKL), secreted by human embryonic trophoblasts and maternal decidual stromal cells (DSCs), polarizes dM*φ* toward a M2 phenotype. This polarization is mediated through activation of Akt/signal transducer and activator of transcription 6 (STAT6) signaling, which is associated with the upregulation of histone H3 lysine-27 demethylase *Jmjd3* and *IRF4* in dM*φ*. Such differentiated dM*φ* can induce a Th2 bias that promotes maternal–fetal tolerance. Impaired expression of RANKL leads to dysfunction of dM*φ in vivo* and increased rates of fetal loss in mice. Transfer of RANK^+^M*φ* reverses mouse fetal loss induced by M*φ* depletion. Compared with normal pregnancy, there are abnormally low levels of RANKL/RANK in villi and decidua from miscarriage patients. These results suggest that RANKL is a pivotal regulator of maternal–fetal tolerance by licensing dM*φ* to ensure a successful pregnancy outcome. This observation provides a scientific basis on which a potential therapeutic strategy can be targeted to prevent pregnancy loss.

Pregnancy constitutes a major challenge to the maternal immune system, which must tolerate fetal alloantigen encoded by paternal genes.^1, 2^ The disturbance of maternal–fetal immune regulation is associated with several complications of human pregnancy, including spontaneous abortion (SA), intrauterine growth restriction (IUGR) and preeclampsia.^3, 4, 5^ Accumulating evidence indicates that decidual macrophages (dM*φ*), the second largest decidual leukocyte population during the first trimester (~20%) following decidual NK cells (dNKs, 50–70%), are involved in several processes required for a successful pregnancy, including trophoblast invasion, as well as tissue and vascular remodeling.^6, 7^ However, the mechanisms responsible for dM*φ* differentiation and polarization at the maternal–fetal interface remain largely unexplored.

Of note, two distinct states of polarized activation of macrophages have been recognized: the classically activated (M1) macrophage phenotype and the alternatively activated (M2) macrophage phenotype.^8, 9, 10^ Bacterial moieties such as LPS and TH1 cytokine interferon-*γ* (IFN-*γ*) polarize macrophages toward the M1 phenotype. These M1 macrophages are characterized by high interleukin (IL)-12 and IL-23 and low IL-10 production, and accordingly can kill intracellular microorganisms and induce Th1 immunity. In contrast, M2 polarization was originally discovered as a response to the Th2 cytokines IL-4 and IL-13, the anti-inflammatory cytokine IL-10, M-CSF, glucocorticoids and immune complexes. They generally share characteristics such as high IL-10 and low IL-12 and IL-23 production, anti-inflammatory and tissue remodeling properties, and scavenging of apoptotic cells and debris, and therefore have been considered to be important regulators of the immune response.

The dM*φ* were classified as resembling an M2 phenotype.^11^ However, there is still ambiguity with regard to the distinct functions of the dM*φ* subset.^4, 6, 12^ Recent research has revealed that first-trimester dM*φ* can be divided into two distinct subsets, CD209^+^ and CD209^−^dM*φ*.^6, 12^ In comparison with CD209^−^dM*φ*, CD209^+^dM*φ* express high levels of the scavenger receptor CD163, the phagocytic receptors CD206 and CD304, and the CD209 ligand ICAM-3, and low levels of CD11c, which are associated with spiral arteriole remodeling.^6^

Receptor activator of NF-*κ*B ligand (TNFSF11, also known as RANKL) and its tumor necrosis factor (TNF)-family receptor RANK are essential regulators of osteoclast differentiation and thereby fundamental aspects of bone physiology, bone remodeling,^13, 14^ lymph node formation,^15^ establishment of thymic microenvironment,^16^ mammary gland development during pregnancy^17, 18^ and bone metastasis of cancer.^19^ Osteoprotegerin (OPG) is a decoy receptor for RANKL. By binding RANKL, OPG blocks the RANKL–RANK interaction. Osteoclasts are derived from monocyte/macrophage precursors.^13^ However, the role of RANKL in inducing macrophage differentiation and functional regulation at the maternal–fetal interface is largely unknown.

In this study, we investigated the effect of RANKL from human embryonic trophoblasts and maternal DSCs on dM*φ* differentiation and maternal–fetal immune tolerance, and we analyzed the relationship of RANKL production at the interface with miscarriage.

## Results

### The crosstalk between fetus and mothers leads to high levels of RANKL/RANK expression at the maternal–fetal interface

**Figure 1:**
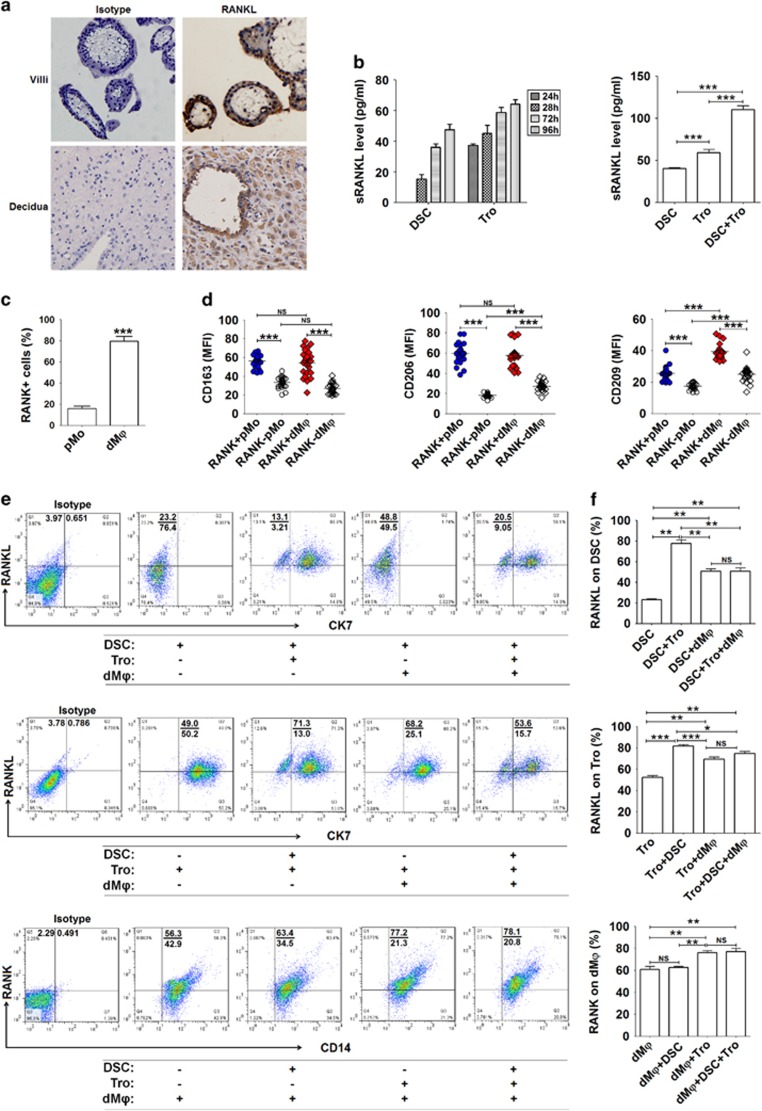
The crosstalk between fetus and mothers leads to high levels of RANKL/RANK expression at the maternal–fetal interface. (**a**) RANKL expression in villi and decidua of normal pregnancy (*n*=12) by immunohistochemistry. Original magnification: × 200. (**b**) RANKL secretion by primary trophoblasts (1 × 10^5^ cells per well) and DSCs (1 × 10^5^ cells per well) (*n*=6) from normal pregnant women by ELISA after culture for 24–96 h (left). RANKL production by trophoblasts alone, DSCs alone and the co-culture of trophoblasts and DSCs (*n*=6) for 48 h (right). (One-way ANOVA). (**c**) We isolated primary PBMCs from peripheral blood (*n*=6) and DLC from deciduas of normal pregnant women (*n*=6), and then analyzed RANK expression on pMo and dM*φ* from normal pregnant women by labeling with anti-CD14, RANK and CD45 antibodies. (Student’s *t*-test). (**d**) Further analysis of the phenotype of RANK^+^ and RANK^−^ pMo and dM*φ* from normal pregnant women (*n*=24) by FCM. (One-way ANOVA). (**e** and **f**) Trophoblasts, DSCs and/or dM*φ* (*n*=6) were co-cultured at a 1 : 1 : 1 ratio for 48 h, and then RANKL expression on CK7^+^ trophoblasts and CK7^−^ DSCs, and RANK on CD14^+^ dM*φ* were evaluated by FCM, respectively. (One-way ANOVA). Tro: human trophoblasts. pMo: human peripheral blood monocytes; dM*φ*: human dM*φ*. Data are expressed as the mean±S.E.M. *P<0.05, **P<0.01 and ***P<0.001. NS: no statistical difference

To investigate the role of RANKL/RANK signaling at the maternal–fetal interface, we first analyzed the expression of RANKL and found that both embryonic trophoblasts from villi and maternal DSCs from decidua are positive for RANKL in human first-trimester pregnancy (Figures 1a and b). As observed by immunohistochemistry, RANKL expression was located both in cell membrane and cytoplasm (Figure 1a). Similar results for RANKL expression levels were obtained by ELISA and flow cytometry (FCM) of the isolated trophoblasts and DSCs. In comparison with DSCs, trophoblasts secreted more soluble RANKL (sRANKL) and expressed higher level of membrane RANKL (mRANKL), and the co-culture of trophoblasts and DSCs produced higher levels of sRANKL (Figure 1b).

To identify target cells of RANKL at the maternal–fetal interface, we analyzed the expression of RANK in decidual leukocyte cells (DLCs) primarily isolated from human decidual tissue. Among DLCs, the percentage of RANK^+^ cells in CD45^+^CD14^+^dM*φ*, as well as decidual NKT and CD3^+^T cells was ~80% (Supplementary Figure 1a), which was 4.45-fold higher than that of CD14^+^monocyte cells from peripheral blood (pMo) (Figure 1c).

Interestingly, further phenotypic analysis showed that both RANK^+^pMo and RANK^+^dM*φ* expressed higher levels of M2-polarized macrophages markers (10) (the scavenger receptor CD163, the phagocytic receptors CD206 and CD209) and M1 co-stimulatory molecules CD80, CD86, HLA-DR, CD11c and cytokine IL-12p40, compared with RANK^−^cells (Figure 1d and Supplementary Figure 1b). These significant differences in M2 phenotype between RANK^+^pMo and RANK^−^pMo was maintained and even augmented in dM*φ*. Conversely, the M1 advantages possessed by RANK^+^pMo gradually weakened in RANK^+^dM*φ*. In comparison with RANK^−^dM*φ*, the expression pattern of RANK^+^ in dM*φ* suggests that RANK signaling may regulate the differentiation and function of dM*φ*.

Embryonic extravillous trophoblasts (EVT) are in direct contact with maternal DSCs and DLCs, and the interaction between these cell subsets has an important role in maintaining maternal–fetal tolerance, which is required for a successful pregnancy.^3^ To investigate this intercellular crosstalk, we co-cultured primary human trophoblasts, DSCs and dM*φ* (T+D+M). FCM analysis revealed markedly increased expression of RANKL in trophoblasts and DSCs and RANK on dM*φ* in the T+D+M co-culture (Figures 1e and f). The percentage of RANK^+^dM*φ* was reduced to 60% after culture alone for 48h. The presence of embryonic trophoblasts in the absence of DSCs led to a frequency of 80% RANK^+^dM*φ* (Figures 1e and f). Taken together, these data suggest that embryonic trophoblasts may have a crucial role in the regulation of maternal dM*φ* differentiation and function through the RANKL–RANK interaction.

### RANKL from trophoblasts and DSCs triggers M2 differentiation of dM*φ* and Th2 bias

**Figure 2:**
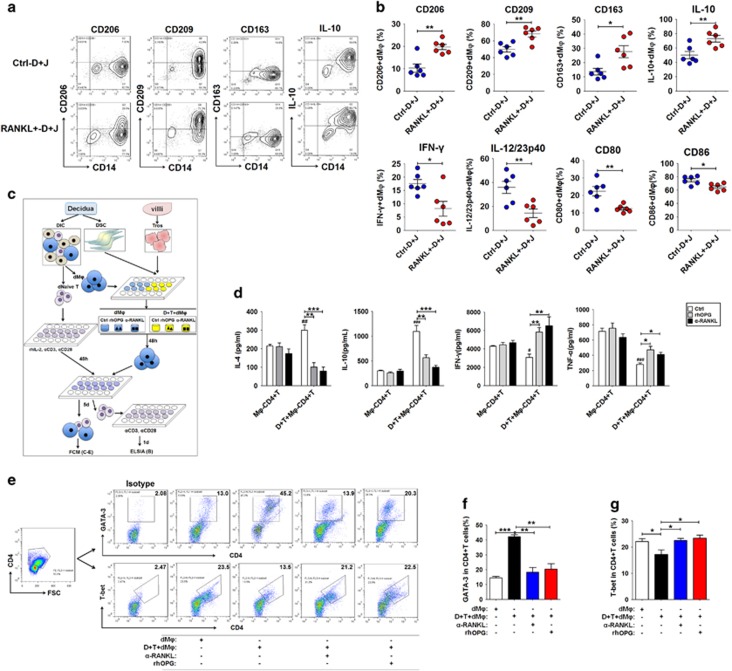
RANKL from trophoblasts and DSCs triggers M2 differentiation of dM*φ* and Th2 bias. (**a** and **b**) We co-cultured dM*φ* (*n*=6) with RANKL-overexpressed or control DSCs and JEG-3 cells at a 1 : 1 : 1 ratio for 48h, and then the expression levels of CD206, CD209, CD163, IL-10, CD80, CD86, IFN-*γ* and IL-12/23p40 were assessed in dM*φ*. (Student’s *t*-test). (**c-g**) After 48 h of culture with or without trophoblasts and DSCs and treatment with or without recombinant human OPG protein (rhOPG, 100 ng/ml) or anti-human RANKL neutralizing antibody (*α*-RANKL, 5 *μ*g/ml), CD14^+^ dM*φ* (*n*=6) were collected and co-cultured with autologous decidual naive T cells at ratios of 1 : 1 (**c**). The decidual naive T cells had been previously activated with anti-CD3 (5 *μ*g/ml), anti-CD28 (1 *μ*g/ml) and rhIL-2 (20 U/ml) for 3 days, and then collected. After 5 days of co-culture, the expression of GATA-3 and T-bet in CD4^+^T cells (**e-g**) were analyzed by FCM; alternately, these CD4^+^T cells were collected and reactivated with anti-CD3 and anti-CD28 alone for another 24 h, and then the secretion levels of IL-4, IL-10, TNF-*α* and IFN-*γ* (**d**) were analyzed by ELISA. (One-way ANOVA). dM*φ*: human dM*φ*; Ctrl-D+J: control DSCs and JEG-3 cells; RANKL^+^D+J: RANKL-overexpressed DSCs and JEG-3 cells; M*φ*-CD4+T: co-culture of ctrl dM*φ* and naive T cells; D+T+CD4+T: co-culture of dM*φ* pre-cultured with DSCs and trophoblasts and naive T cells. Data are expressed as the mean±S.E.M. *P<0.05, **P<0.01 and ***P<0.001. ^#^*P*<0.05, ^##^*P*<0.01 and ^###^*P*<0.001 *versus* ctrl M*φ*-CD4+T

To investigate the potential effect of RANKL on dM*φ*, we directly co-cultured the purified CD14^+^dM*φ* with RANKL-overexpressed DSCs and JEG-3 cells (human placental choriocarcinoma cell line) (RANKL^+^D+J). Compared with control DSCs and JEG-3 cells (Ctrl-D+J), RANKL^+^D+J resulted in the elevation of CD206, CD209, CD163 and IL-10 and the decline of IFN-*γ*, IL-12/23p40, CD80 and CD86 in CD14^+^dM*φ* (Figures 2a and b). In contrast, blocking the RANKL–RANK interaction with a neutralizing antibody against RANKL (*α*-RANKL) or OPG protein could reverse the expression of CD80 and CD86 and the release of IL-10, IL-12p40 and IL-23 induced by RANKL of trophoblasts and DSCs (Supplementary Figure 2).

RANKL participates in the regulation of monocyte migration by inducing the secretion of chemokine, such as CCL22 and CCL2.^20, 21^ However, current results show that RANKL was not involved in the regulation of the M2-recruited chemokines CCL17, CCL22 and CCL24 and the M1-recruited chemokines CXCL9 and CXCL10 of dM*φ* (data not shown). We have previously reported that RANKL stimulates DSCs to secrete the chemokine CCL2.^22^ Our study suggests that RANKL may recruit peripheral monocyte to decidua, and further polarize them toward M2 dM*φ*.

Evidence suggests that the RANKL–RANK interaction increases macrophage/dendritic cell (DC) survival and enhances the induction of T-cell response.^23, 24, 25^ However, published evidence shows that RANKL-mediated modulation of DCs in mucosal tissues increases the number of CD4^+^CD25^+^ regulatory T (Treg) cells and promotes immunosuppressive activity toward foreign antigens, such as food or commensal bacteria in the intestines.^26, 27, 28^ However, the molecular mechanism underlying RANKL on M*φ*s and DCs remains unclear. We further investigated the regulation of these RANKL-instructed dM*φ* on the differentiation of decidual naive T cells. After pre-culture with trophoblasts and DSCs, we collected dM*φ*, and then co-cultured them with decidual naive T cells for 5 days (Figure 2c). We observed that either *α*-RANKL or OPG abolished the stimulatory effect on the IL-10 and Th2 transcription factor GATA-3 and the inhibitory impact on tumor necrosis factor-*α* (TNF-*α*) and Th1 transcription factor T-bet in CD4^+^ T cells mediated by dM*φ* pre-treated with trophoblasts and DSCs (D+T-dM*φ*) (Figures 2d and g). In addition, blocking RANKL in the T+D+M co-culture further inhibited IL-4 secretion but stimulated IFN-*γ* production of CD4^+^T cells (Figures 2d and g). However, RANKL-instructed dM*φ* had no effect on decidual Treg cell differentiation (data not shown).

As a potent inducer of decidual M2 M*φ*,^6^ the increased IL-10 in dM*φ* and decidual CD4^+^T cells induced by RANKL may further amplify the impact of RANKL on M2 polarization of dM*φ*. These data provide strong evidence that RANKL is expressed at the maternal–fetal interface, and support the presence of a positive regulatory loop between trophoblasts and dM*φ* to induce maternal–fetal immune tolerance during pregnancy.

### The effect of RANKL on dM*φ* is dependent on the activation of the Akt/STAT6-Jmjd3/IRF4 signaling pathway

**Figure 3:**
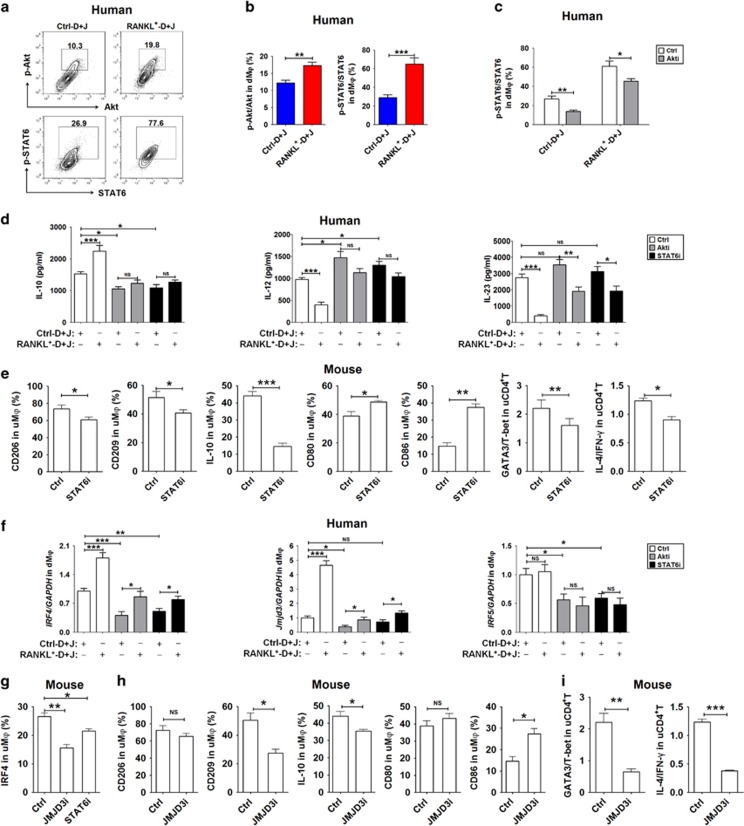
RANKL induces dM*φ* differentiation by activating the Akt/STAT6-Jmjd3/IRF4 signaling pathway. (**a-c**) dM*φ* were co-cultured with RANKL^+^D+J or Ctrl-D+J at a 1 : 1 : 1 ratio, and treated with or without 10 *μ*M Akt signaling inhibitor (Akti) Ly294002 for 24 h. The intracellular phosphorylation level of Akt and or STAT6 in dM*φ* (*n*=5) was then analyzed by FCM. (**d**) The secretion level of IL-10, IL-12p40 and IL-23 in the co-culture of dM*φ* and RANKL^+^-D+J or Ctrl-D+J (at a 1 : 1 : 1 ratio), which was treated with or without Akti (10 *μ*M) or the STAT6 signaling inhibitor (STAT6i) AS1517499 (21 nM) for 48 h. (One-way ANOVA). (**e**) After intraperitoneal injection of STAT6i (200 *μ*l at the concentration of 2 mg/kg) in pregnant C57BL/6 mice (*n*=5 mice per group) at day 4, the levels of CD206, CD209, IL-10, CD80 and CD86 in uM*φ*, and the ratios of GATA-3 to T-bet and IL-4 to IFN-*γ* in uCD4^+^T cells at day 10 were detected by FCM. (Student’s *t*-test). (**f**) The transcription levels of *IRF4, Jmjd3* and *IRF5* in dM*φ* treated as described in Figure 3d. (One-way ANOVA). (**g**) After intraperitoneal injection of STAT6i or the Jmjd3 selective inhibitor (JMJD3i, 200 *μ*l at a concentration of 4.48 mg/kg) GSK J4 HCl in pregnant C57BL/6 mice (*n*=5 mice per group) at day 4, the IRF4 level in uM*φ* cells at day 10 was detected. (**h** and **i**) After intraperitoneal injection of JMJD3i in pregnant C57BL/6 mice (*n*=5 mice per group) at day 4, the levels of CD206, CD209, IL-10, CD80 and CD86 in uM*φ*, and the ratios of GATA-3 to T-bet and IL-4 to IFN-*γ* in uCD4^+^T cells at day 10 were detected by FCM. (Student’s *t*-test). dM*φ*: human dM*φ*; uM*φ*: mouse uterus macrophages. Data are expressed as the mean±S.E.M. *P<0.05, **P<0.01 and ***P<0.001

Of note, mRANKL or sRANKL cleaved by matrix metalloproteinases (MMPs) or a disintegrin and metalloproteases (ADAMs) binds RANK and then mainly activates the nuclear factor kappa-light-chain-enhancer of activated B cells (NF-*κ*B) pathway to control osteoclastogenesis through adaptor molecules such as TRAFs and Gab2,^29, 30, 31^ thus regulating osteoimmunology by pro- and anti-inflammatory effects on the immune system.^32^ To identify the downstream pathway through which RANKL drives M2 polarization during pregnancy, we assessed the impact of RANKL on different signaling pathways in dM*φ*. T+D+M led to changes in the phosphorylation of several signaling pathways such as Akt, NF-*κ*Bp65 and c-Jun N-terminal kinases (JNK) (data not shown), suggesting that several pathways should be involved in the functional regulation on dM*φ*. Notably, *α*-RANKL or OPG specifically inhibited the activation of Akt and STAT6 (a master regulator of M2 genes in mice downstream of IL-4R)^33, 34, 35^ in dM*φ* (data not shown). Culture with RANKL^+^-D+J gave rise to the increased level of Akt and STAT6 phosphorylation in dM*φ* compared with the culture with Ctrl-D+J (Figures 3a and b). The Akt signal inhibitor (Akti) LY294002 partly reversed the level of STAT6 phosphorylation (Figure 3c).

Compared with the control, the release of IL-10 and IL-12p40 and IL-23 by dM*φ* increased and decreased, respectively, under co-culture with RANKL^+^-D+J, and these effects could be clearly impaired by Akti or STAT6 inhibitor (STAT6i) AS1517499^35^ treatment (Figure 3d). Interestingly, treatment with STAT6i resulted in M1 differentiation and Th1 bias in the mice uterus *in vivo* (Figure 3e). Therefore, these data suggest that RANKL induces M2 differentiation by activating the Akt/STAT6 signaling pathway.

It has been reported that Jmjd3 regulates M2 polarization by inhibiting the transcription of typical M1-associated genes and inducing IRF4.^36, 37, 38^ Unlike IRF5,^39^ IRF4 has been recognized as an essential transcription factor for M2 polarization and the expression of M2 signature genes such as *Arg1, Ym1 and Fizz1* in mice.^40^ We investigated the effects of RANKL on these transcription factors. Blocking RANKL resulted in a marked decrease in *Jmjd3* and *IRF4* but not *IRF5* transcription in the co-culture of D+T-dM*φ* (Supplementary Figure 3). Conversely, there was an increase in the transcription of *Jmjd3* and *IRF4* in RANKL^+^-D+J-dM*φ*, and both Akti and STAT6i treatment could partially abrogate these effects on *Jmjd3* and *IRF4* induced by RANKL *in vitro* (Figure 3f). Similarly, both Jmjd3 selective inhibitor (JMJD3i) GSK J4 HCl and STAT6i treatment could downregulate IRF4 expression in uM*φ* (Figure 3g). Furthermore, treatment with JMJD3i also led to M1 differentiation (Figure 3h) and a Th1 bias (Figure 3i) in mouse uterus. Taken together, these results place RANKL upstream in the Akt/STAT6-Jmjd3/IRF4 signaling cascade involved in M2 polarization of dM*φ*.

### Downregulation of RANKL expression leads to murine decidual M*φ* dysfunction and fetal loss

**Figure 4:**
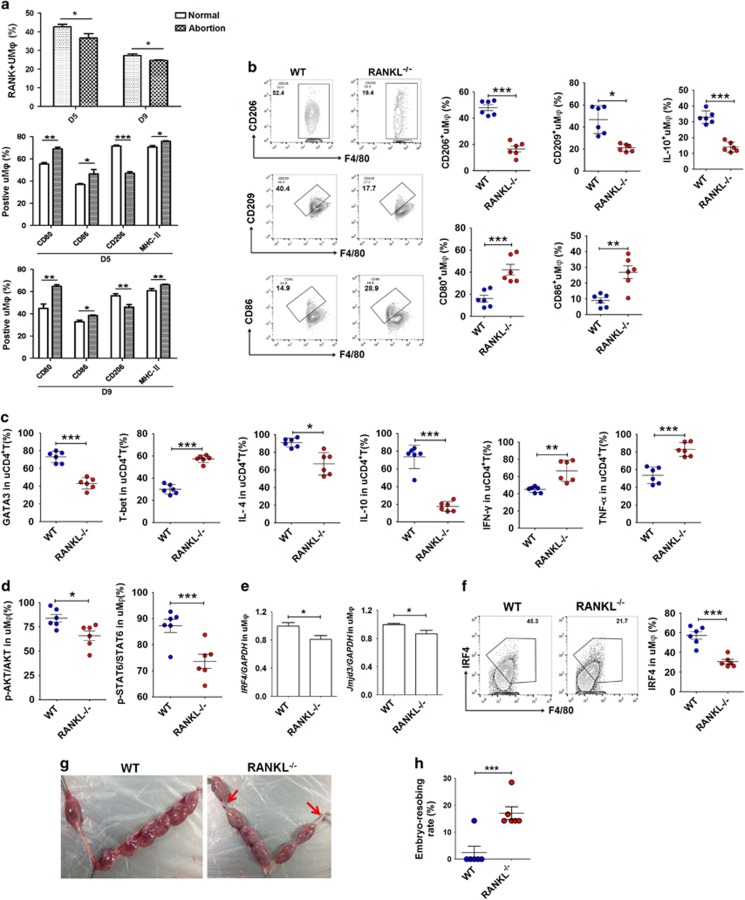
Absence of RANKL expression leads to mouse uM*φ* dysfunction and fetal loss. (**a**) RANK expression on uM*φ* from CBA/J♀ × DBA/2♂ matings (the abortion-prone model) and CBA/J♀ × BALB/c♂ matings (normal pregnancy model) at days 5 and 9 of gestation (*n*=6 mice per group). Moreover, the expression of CD80, CD86, CD206 and MHCII on F4/80^+^uM*φ* from CBA/J♀ × DBA/2♂ matings (the abortion-prone model) and CBA/J♀ × BALB/c♂ matings (normal pregnancy model) at days 5 and 9 of gestation (*n*=6 mice per group); (adjusted *t*-test). (**b**) FCM analysis of CD206, CD209, IL-10, CD80 and CD86 in uM*φ* of wild-type and RANKL knockout pregnant mice at day 10 (*n*=6 mice per group); (Student’s *t*-test). (**c**) FCM analysis of GATA-3, T-bet, IL-4, IL-10, IFN-*γ* and TNF-*α* in uCD4^+^T cells of WT and RANKL^−/−^ pregnant mice at day 10 (*n*=5 mice per group); (Student’s *t*-test). (**d**) FCM analysis of the phosphorylation level of Akt and STAT6 in uM*φ* cells of WT and RANKL^−/−^ pregnant mice at day 10 (*n*=6 mice per group); (Student’s *t*-test). (**e**) uM*φ* were isolated from mouse uterus (*n*=20 mice per group) from WT and RANKL^−/−^ mice at day 10 of gestation by MACS, and then used to analyze the transcription of *Jmid3* and *IRF4* in uM*φ*. (Student’s *t*-test). (**f**) FCM analysis of IRF4 levels in uM*φ* cells of WT and RANKL^−/−^ pregnant mice at day 10 (*n*=6 mice per group); (Student’s *t*-test). (**g** and **h**) The embryo absorption rate in WT and RANKL^−/−^ pregnant mice (*n*=6 mice per group) was determined on day 14 of gestation. Fetal loss sites could be identified as hemorrhagic spots and necrosis (red arrows, left); (adjusted *t*-test). uM*φ*: M*φ* from mouse uterus; uCD4^+^T cells: CD4^+^T cells from mouse uterus; Normal: normal pregnant mouse model; Abortion: abortion mouse model. D5: day 5 of gestation; D9: day 9 of gestation. WT: wild-type pregnant mice; RANKL^−/−^: RANKL knockout pregnant mice. Data are expressed as the mean±S.E.M. *P<0.05, **P<0.01 and ***P<0.001

In comparison with non-pregnant mice, uterine M*φ* (uM*φ*) from pregnant mice had a high level of RANK (Supplementary Figure 4). To further explore the role of absent RANKL at the maternal–fetal interface during the differentiation of M*φ in vivo*, we first evaluated RANKL/RANK expression in the uterus in normal pregnancy and abortion-prone matings. In CBA/J × DBA/2 matings as an abortion-prone model, decreased RANK expression was detected in F4/80^+^uM*φ* on gestational days 5 and 9, compared with CBA/J × BALB/c matings as a mouse model of normal pregnancy (Figure 4a).

Interestingly, uM*φ* in mice are divided into two subsets: F4/80^+^MHCII^hi^ and F4/80^+^MHCII^lo^. These two subsets differentially express *CD163* and *Mrc1*, which may represent mouse analogs of CD209^−^ and CD209^+^dM*φ*.^4, 41^ Here, a characteristic M1 rather than M2 phenotype was also observed in uM*φ* from CBA/J × DBA/2 abortion-prone matings (Figure 4a). Similarly, uM*φ* in RANKL knockout (RANKL^−/−^) pregnant mice had low levels of CD206, CD209 and IL-10, and high levels of CD80 and CD86 compared with wild-type (WT) pregnant mice (Figure 4b). In comparison with WT group, there was a Th1 bias in the uterus of RANKL^−/−^pregnant mice (Figure 4c). Furthermore, there were low levels of Akt and STAT6 phosphorylation, Jmjd3 and IRF4 in uM*φ* of RANKL^−/−^pregnant mice compared with WT group (Figures 4d–f).

To investigate the influence of RANKL/RANK signaling on outcome of pregnancy *in vivo*, we evaluated embryo-absorbing level between RANKL^−/−^ and WT pregnant mice. The embryo-absorbing site could be macroscopically distinguished as hemorrhagic spots and necrosis at late gestation (gestational day 14, Figure 4g). The RANKL^−/−^mice were more susceptible to fetal loss than WT mice (Figures 4g and h). These findings provide evidences of a key role of RANKL in the regulation of dM*φ* differentiation and function, promoting maternal–fetal tolerance to support normal pregnancy. Abnormal suppression of RANKL expression contributes to uM*φ* dysfunction and fetal loss *in vivo*.

### Adoptive transfer of RANK^+^M*φ* relieves murine embryo absorption induced by M*φ* depletion

**Figure 5:**
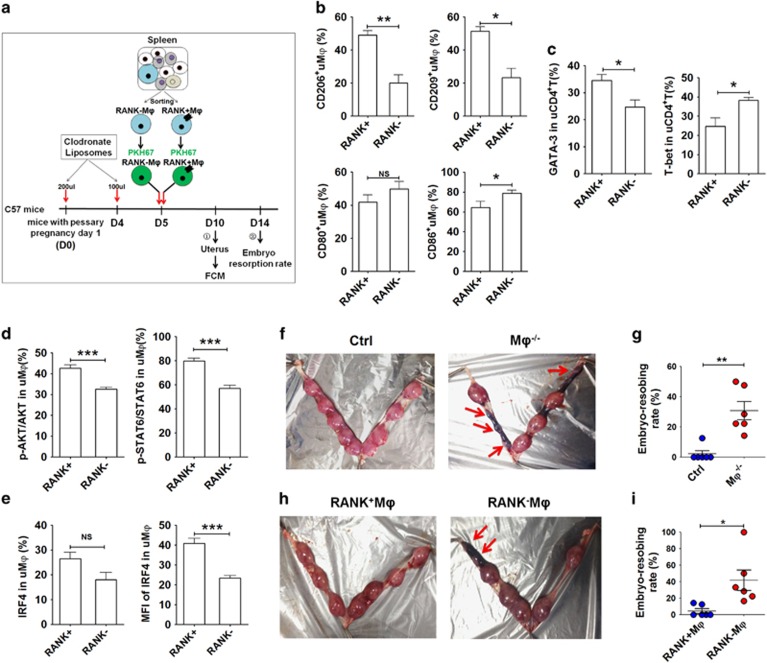
Adoptive transfer of RANK^+^ M*φ* relieves mouse embryo absorption induced by M*φ* depletion. (**a**) RANK^+^ and RANK^−^ M*φ*s were isolated from mouse spleen, labeled with PKH-67, and then transferred to M*φ*-depleted pregnant mice at day 5 of gestation. The uterus was then collected and analyzed by FCM at day 10, and the embryo resorption ratio was observed at day 14. (**b**) FCM analysis of CD206, CD209, CD80 and CD86 in PKH-67-uM*φ* with PKH-67-RANK^+^M*φ* and PKH-67-RANK^−^M*φ* transfer at day 10 (*n*=5 mice per group). (Student’s *t*-test). (**c**) FCM analysis of GATA-3 and T-bet in PKH-67-uM*φ* with PKH-67-RANK^+^M*φ* and PKH-67-RANK^−^M*φ* transfer at day 10 (*n*=5 mice per group); (Student’s *t*-test). (**d**) FCM analysis of the phosphorylation level of Akt and STAT6 in uM*φ* cells with PKH-67-RANK^+^M*φ* and PKH-67-RANK^−^M*φ* transfer at day 10 (*n*=5 mice per group); (Student’s *t*-test). (**e**) FCM analysis of the percentage and median fluorescence intensity (MFI) of IRF4 in uM*φ* cells with PKH-67-RANK^+^M*φ* and PKH-67-RANK^−^M*φ* transfer at day 10 (*n*=5 mice per group); (Student’s *t*-test). (**f** and **g**) The embryo absorption rate in ctrl pregnant C57BL/6 mice and pregnant C57BL/6 mice with M*φ* depletion (*n*=6 mice per group) were counted on day 14 of gestation (adjusted *t*-test). (**h** and **i**) The embryo absorption rate in pregnant C57BL/6 mice with M*φ* depletion (*n*=6 mice per group) was counted on day 14 of gestation, after adoptive transfer of RANK^+^M*φ* and RANK^−^M*φ* at day 5 (adjusted *t*-test). RANK^+^: adoptive transfer of PKH-67-RANK^+^M*φ*; RANK^−^: adoptive transfer of PKH-67-RANK^−^M*φ*; uM*φ*: M*φ* from mouse uterus. Data are expressed as the mean±S.E.M. *P<0.05, **P<0.01 and ***P<0.001

The depletion of M*φ* in pregnant mice using Clodronate Liposomes (Supplementary Figures 5a and b) led to a significant decrease in RANKL in uterine DSCs (uDSCs) and placental trophoblasts (pTros) (Supplementary Figure 5c), suggesting that dM*φ*s are involved in the maintenance of high levels of RANKL at the maternal–fetal interface. To provide insight into the role of RANKL-instructed dM*φ* in maternal–fetal immune regulation and pregnancy outcome *in vivo*, we evaluated the effect of dM*φ* depletion and adoptive transfer of RANK^+^M*φ* on these processes. Next, we isolated RANK^+^ and RANK^−^M*φ*s from mouse spleen and observed that these RANK^+^M*φ*s, like human RANK^+^pMo, had high levels of M1 and M2 phenotype molecules (Supplementary Figure 6a). To investigate the process of RANK^+^M*φ* differentiation in the uterus *in vivo*, we labeled these RANK^+^ and RANK^−^M*φ*s with PKH-67 and transferred them to M*φ*-deleted pregnant mice (Figure 5a and Supplementary Figure 6b). RANK^+^M*φ*s with high CCR2 were preferentially recruited to the uterus (Supplementary Figure 6c). In comparison with the PKH-67-RANK^−^M*φ* transferred group, PKH-67-RANK^+^M*φ* recruited to uterus presented high levels of CD206 and CD209, and low levels of CD86 and similar levels of CD80 (Figure 5b). In addition, the transfer of PKH-67-RANK^+^M*φ* led to a Th2 bias in mouse uterus (Figure 5c), an increase in Akt and STAT6 activation (Figure 5d), and a high level of IRF4 (Figure 5e) in uM*φ*.

Subsequently, we observed that M*φ* depletion caused a significant increase in fetal loss (Figures 5f and g). To further identify the role of RANKL-instructed M*φ* in ameliorating fetal loss, we transferred RANK^+^ or RANK^−^M*φ* to M*φ*-deleted pregnant mice and found that adoptive transfer of RANK^+^M*φ* could significantly relieve the murine embryo absorption induced by M*φ* depletion (Figures 5h and i).

### The suppression of RANKL/RANK expression and dM*φ* dysfunction in human miscarriage

**Figure 6:**
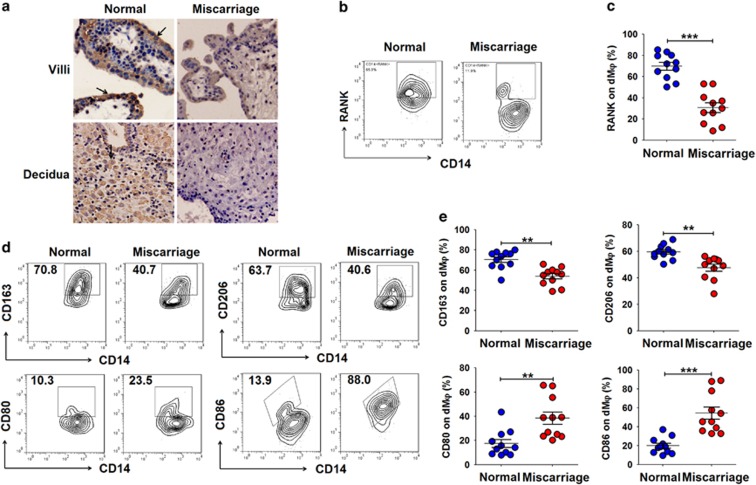
There are low levels of RANKL/RANK at the maternal–fetal interface during miscarriage. (**a**) Immunohistochemistry analysis of RANKL expression in villi and deciduas from women with normal pregnancy (*n*=12) or miscarriage (*n*=12) during the first trimester. RANKL expression was localized to the cell membrane and the cytoplasm (arrows) in the deciduas and villi. Original magnification: × 200. (**b** and **c**) FCM analysis of the percentage of RANK^+^ dM*φ* from women with normal pregnancy (*n*=11) or miscarriage (*n*=11) during the first-trimester. (**d** and **e**) FCM analysis of the percentage of CD163^+^, CD206^+^, CD80^+^ and CD86^+^dM*φ* from women with normal pregnancy (*n*=11) or miscarriage (*n*=11) during the first trimester. Normal: normal pregnant women; Miscarriage: SA women. Data are expressed as the mean±S.E.M. **P<0.01 and ***P<0.001 (Student’s *t*-test)

The imbalance of maternal–fetal immunoregulation has been previously reported in pregnancy complications^3, 4, 5^ such as miscarriage, preeclampsia and IUGR. Therefore, we evaluated RANKL/RANK expression in the tissue from patients with miscarriage during the first trimester. In comparison with normal pregnancy, we observed a decrease in RANKL expression in trophoblasts and DSCs (Figure 6a), as well as reduced RANK expression on CD14^+^dM*φ* (Figures 6b and c) in miscarriage, accompanied by a decreased frequency of dM*φ* with an M2 phenotype and an increase in the M1 phenotype (Figures 6d and e). Taken together, the suppression of RANKL/RANK signaling may result in dM*φ* dysfunction and further trigger miscarriage during the first trimester.

## Discussion

**Figure 7:**
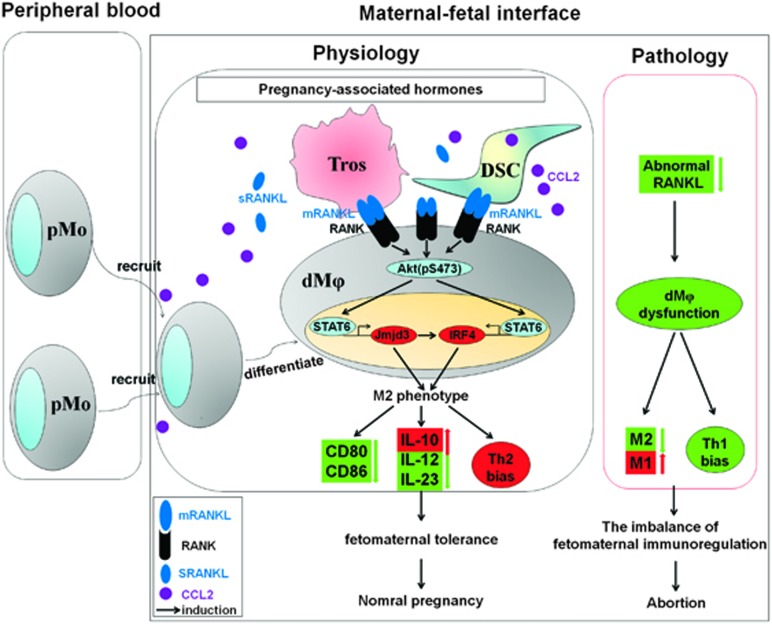
RANKL-mediated harmonious dialog between the fetus and mother guarantees a smooth gestation by inducing decidual M2 macrophage polarization. Together with blastocyst implantation during normal pregnancy, PAHs trigger ESCs to differentiate into DSCs, further inducing high levels of RANKL expression and CCL2 release. The latter recruits more peripheral monocytes to the decidua. Embryonic trophoblasts that are deeply implanted in decidua can closely contact DSCs and DLCs. This dialog not only further increases RANKL expression in trophoblasts and DSCs, but it also enhances the sensitivity of RANK to RANKL by upregulating RANK expression on M*φ*. Subsequently, the RANKL–RANK interaction drives M*φ* to M2 differentiation (low expression of CD80 and CD86, high secretion of IL-10, and low level of IL-12 and IL-23) by activating Akt/STAT6-Jmjd3/IRF4 signaling. After education, these M*φ*s will create and maintain a maternal immune tolerance microenvironment (increase in Th2 and decrease in Th1). After the development of abnormally low RANKL expression at the maternal–fetal interface, the dysfunction of M*φ*, the imbalance of maternal–fetal immune regulation and then abortion will occur. Tro: trophoblasts

Collectively, we have demonstrated that RANKL derived from embryonic trophoblasts and maternal DSCs drives dM*φ* polarization toward an M2 phenotype by activating Akt/STAT6 signaling and enhancing the transcription of *IRF4* and *Jmjd3*; finally, it contributes to the formation and maintenance of maternal–fetal tolerance by further inducing Th2 bias (Figure 7). This maternal–fetal dialog mediated by RANKL guarantees a smooth gestation by creating a tolerant microenvironment. The process of maternal–fetal tolerance formation is not only derived from a maternal behavior for immune adaptation to pregnancy but also, most importantly, the fetus can instruct the mother’s immune system to adapt to it.

In comparison with the peripheral, the M2 phenotype advantage of RANK^+^dM*φ* becomes more prominent in the decidua. The levels of M1 phenotype markers in RANK^+^dM*φ* are also higher than those in RANK^−^dM*φ*, but still very low, similar to the level of RANK^−^ pMo. Similarly, it has been reported that both CD209^+^ and CD209^−^dM*φ* stimulate the release of proinflammatory cytokines such as IL-6 and TNF-*α* after LPS stimulation *in vitro*.^12^ These data emphasize the complexity of dM*φ* biology. During normal pregnancy, the M2 advantage of dM*φ* at the maternal–fetal interface is relative and mainly depends on the local microenvironment. This advantage may be disrupted by intrauterine infection and lead to an M1 advantage to limit infection. The expression of proinflammatory molecules in dM*φ* may align more with the theory that immune activation is required to facilitate trophoblast invasion and implantation, as well as the establishment of fetal–maternal tolerance during the first trimester.

The human maternal–fetal interface is characterized by intimate contact between the maternal decidua and extravillous cytotrophoblast cells that invade the decidua. Trophoblasts can influence the maternal immune system during pregnancy by expressing soluble and cell surface molecules, such as HLA-G,^42, 43^ IDO^44^ and anti-inflammatory cytokines.^45, 46, 47^ These molecules limit the proliferation and activation of T cells, antigen-presenting cells and NK cells in decidua. In our present study, we found that the crosstalk between embryonic trophoblasts and maternal DSCs and dM*φ* contributes to the accumulation of RANKL expression at the maternal–fetal interface. RANKL expressed by trophoblasts and DSCs induces M2 differentiation of dM*φ* and further drives the Th2 bias, suggesting that RANKL/RANK signaling has a critical role in dM*φ* differentiation and maternal–fetal tolerance. It is noteworthy that trophoblasts upregulate RANK expression specifically on dM*φ*. These findings further highlight the core role of trophoblasts in dM*φ* differentiation regulation. Rather than traditional NF-*κ*B signaling under the RANKL/RANK axis, we found that Akt/STAT6-Jmjd3/IRF4 signaling is required for M2 differentiation of dM*φ* induced by RANKL at the maternal–fetal interface *in vitro* and *in vivo*. Further studies should clarify the molecular mechanisms by which RANKL specifically activates Akt/STAT6 signaling in dM*φ*.

In human pregnancy, embryo implantation in the receptive endometrium triggers a series of responses collectively called decidualization. During decidualization, endometrial stromal cells (ESCs) undergo steroid hormone-dependent proliferation and differentiation into decidual cells.^48^ Interestingly, pregnancy-associated hormones (PAHs, such as estrogen and prolactin) upregulate RANKL and RANK levels, downregulate OPG expression, and further affect osteoclastogenesis at distinct stages of development.^49, 50^ RANKL/RANK system also controls the incidence and onset of progestin-driven breast cancer and physiological thermoregulation in females under the control of sex hormones.^51, 52^ Therefore, high levels of PAH during pregnancy may also be one of the important factors leading to such high levels of RANKL/RANK at the maternal–fetal interface.

The decidua has been considered a specialized mucosal wall of the uterus. Research examining other mucosal tissues (skin and intestine) shows that epidermal and Peyer’s patch-derived DCs stimulated with RANKL induce immunosuppressive activity by modulating surface barrier DCs and increasing the expansion and function of Treg cells.^26, 27, 28^ Our results partially echo the immunosuppressive effect of RANKL in the mucosa. This function in the decidua is independent of the regulation of Treg differentiation.

In comparison with normal pregnancy, we observed that RANKL in trophoblasts and DSCs and RANK on dM*φ* in patients with miscarriage were greatly decreased. Moreover, the dM*φ* phenotype during human and mouse pregnancy wastage shows an M1 predominance. RANKL^−/−^mice presented uM*φ* dysfunction and increased fetal loss. This deregulation of uM*φ* supports an inflammatory environment that further triggers abortive processes.^53^ Therefore, our study reveals a novel pathogenic role of abnormal RANKL/RANK signaling at the maternal–fetal interface during SA in humans and mice. Trials conducted *in vivo* also showed that RANKL^−/−^mice had no significant influence on the total number of embryo implantations (data not shown). However, our unpublished data show that either endogenous or exogenous RANKL directly stimulates the proliferation and enhances the invasiveness of human trophoblasts, partially echoing its role in tumor cells.^19^ We propose that the lack of RANKL *in vivo* may result in a decrease in trophoblast proliferation and invasion, but to a certain extent, it will also create a proinflammatory microenvironment. This inflammatory pattern during the initial stage of pregnancy may be conducive to the invasion and implantation of trophoblasts. Therefore, the overall effect of absent RANKL signaling *in vivo* may not affect embryo implantation. However, with advancing pregnancy, abnormally low levels of RANKL will result in miscarriage via the M1 dM*φ*-triggered disorder of maternal–fetal immune tolerance. Therefore, further research is needed to elucidate the cause of low RANKL/RANK expression in miscarriage patients.

In conclusion, as shown in Figure 7, accompanied by the implantation of blastocyst during normal pregnancy, PAH trigger ESCs to differentiate into DSCs and further induce high levels of RANKL expression and CCL2 release. The latter allows the recruitment of peripheral monocytes into decidua. Embryonic trophoblasts that are deeply implanted in decidua are in close contact with DSCs and DLCs. The dialog of these cells not only further increases RANKL expression in trophoblasts and DSCs but also enhances the sensitivity of RANK on dM*φ* to RANKL by upregulating RANK expression. Subsequently, the RANKL–RANK interaction drives dM*φ* to M2 differentiation. After being instructed, these dM*φ* will create and maintain a maternal–fetal tolerant microenvironment. Once the maternal–fetal interface presents abnormal low level of RANKL, dysfunction of dM*φ* and then miscarriage will occur. Therefore, our study provides a potential target molecule, RANKL, for the identification of new strategies to prevent and treat pregnancy complications.

## Materials and methods

### Patient and sample collection

All procedures involving participants in this study were approved by the Human Research Ethics Committee of Obstetrics and Gynecology Hospital, Fudan University (Shanghai, China), and all subjects completed an informed consent to collect tissue samples. First-trimester human peripheral blood was obtained from 41 women with clinically normal pregnancies (age: 27.45±7.21 years; gestational age at sampling: 48.35±7.6 days (mean±S.D.)]), which were terminated for nonmedical reasons. Human villi tissues were obtained from 172 women with clinically normal pregnancies (age: 29.88±6.91 years; gestational age at sampling: 49.17±9.34 days [mean±S.D.]) (Termination for nonmedical reasons), or from 12 women with SA (age: 31.09±4.28 years; gestational age at sampling: 47.95±10.1 days (mean±S.D.)). Decidual tissues were obtained from 135 women with clinically normal pregnancies (age: 27.19±5.61 years; gestational age at sampling: 51.09±8.72 days (mean±S.D.)) (termination for nonmedical reasons) and 23 women with spontaneous miscarriage (age: 31.54±5.71 years; gestational age at sampling: 53.06±5.8 days (mean±S.D.)). All pregnant women were confirmed by ultrasound and blood tests, and the women with spontaneous miscarriage because of endocrine, anatomical, and genetic abnormalities, as well as infection were excluded.

### Cell line

The human placental choriocarcinoma cell line (JEG-3 cells) was purchased from Bank of Cell, Chinese Academy of Sciences, Shanghai, China.

### Mice

RANKL heterozygote mice were obtained from the Jackson Laboratories (Sacramento, CA, USA) and subsequently maintained in the Laboratory Animal Facility of Fudan University (Shanghai, China). RANKL knockout (RANKL^−/−^) mice and wild-type littermates (WT) were obtained by mating of male and female RANKL heterozygote mice. A group of adult female C57BL/6 mice were purchased from the Laboratory Animal Facility of Fudan University and used for this study. They were usually maintained for 2 weeks in the animal facility before use. The Animal Care and Use Committee of Obstetrics and Gynecology Hospital, Fudan University approved all animal protocols.

### Immunohistochemistry

Immunohistological staining was performed as previously described.^46^ Human villi and deciduas were labeled with mouse anti-RANKL Abs (15 *μ*g/ml, MAB626, R&D Systems, Abingdon, UK).

### Antibodies for FCM

For the identification of cell purity, primary trophoblasts and DSCs were stained with phycoerythrin (PE)-conjugated anti-human vimentin (562337; BD Biosciences) and fluorescein isothiocyanate (FITC)-conjugated anti-human cytokeratin (347653; BD Biosciences, San Jose, CA, USA); DLCs were stained with allophycocyanin (APC)-conjugated anti-human CD45 antibody (304012; Biolegend); dM*φ* was stained with FITC-conjugated anti-human CD14 antibody (301804; Biolegend); decidual naive T cells were stained with FITC-conjugated anti-human CD4 antibody (300506; Biolegend) and PE-conjugated anti-human CD45RA antibody (304107; Biolegend, San Diego, CA, USA).

PE-conjugated anti-human RANKL antibody (347504; Biolegend) and PE-conjugated anti-human RANK antibody (FAB683P; R&D Systems) were used to analyze the expression of RANKL on trophoblasts and DSCs, and the expression of RANK on dM*φ*; pMo and or dM*φ* were stained with FITC-conjugated anti-human CD14 antibody (301804), phycoerythrin -cyanine 7 (PE-Cy7)-conjugated CD80 antibody (305218), APC-conjugated CD86 antibody (305412), PE-Cy7-conjugated IL-10 antibody (501420), PE-conjugated IL-12/23p40 antibody (501807), APC-conjugated IFN-*γ* (502511), APC-conjugated CD206 (321110), allophycocyanin-cyanine 5.5 (PerCP/Cy5.5)-conjugated CD209 antibody (330110) and PE-conjugated CD163 antibody (333606) (all from Biolegend). Decidual naive T cells were stained with PE-Cy7-conjugated CD4 antibody (303718), Alexa Fluor 488-conjugated anti-GATA-3 (653808) and PE-conjugated anti-T-bet (644810) (all from Biolegend).

The uSC and pTros were stained with PE anti-mouse RANKL antibody (510005); the uM*φ* was stained with PE anti-mouse RANK antibody (119805), Alexa Fluor 647 anti-mouse F4/80 antibody (122610), PE/Cy7 anti-mouse CD45 antibody (103114), FITC anti-mouse CD11b antibody (101206), FITC anti-mouse CD80 antibody (104706), PE anti-mouse CD86 antibody (105008), FITC anti-mouse CD206 (MMR) antibody (141704), Brilliant Violet 421 (BV421) anti-mouse IL-10 antibody (505021) (all from Biolegend), PE anti-mouse CD209 (DC-SIGN) antibody (12-2091; ebioscience; Thermo Fisher Scientific, Inc., Waltham, MA, USA), PE anti-mouse MHCII antibody (12-5322; ebioscience), and PE/Cy7 anti-IRF4 antibody (25-9858; ebioscience); uCD4^+^T cells were stained with PE/Cy7 anti-mouse CD45 antibody (103114), FITC anti-mouse CD4 antibody (100405), PE anti-mouse IL-4 antibody (504103), BV421 anti-mouse IL-10 antibody (505021), APC anti-mouse IFN-*γ* antibody (505810), PE anti-mouse TNF-*α* antibody (506305) (all from Biolegend), BV421 anti-mouse GATA-3 antibody (563349; BD Biosciences), and PE anti-mouse T-bet antibody (561268; BD Biosciences).

For intracellular staining of signaling molecules in dM*φ* or uM*φ*, cells were permeabilized (Cytofix/Cytoperm kit; BD Biosciences) and incubated with Alexa Fluor 647-conjugated Akt (pS473) antibody (561670) and Alexa Fluor 488-conjugated Stat6 (pY641) antibody (612600 for dM*φ*; 558243 for uM*φ*) (Phosflow antibodies were from BD Biosciences).

### Isolation and culture of human trophoblasts, DSCs and DLCs

The villi and decidua tissues from the first-trimester pregnancy were put immediately into ice-cold Dulbecco’s modified Eagle’s medium (DMEM high d-glucose; Gibco, Grand Island, NY, USA), transported to the laboratory within 30 min after surgery and washed in Hank’s balanced salt solution for isolation of human trophoblasts from villi, and DSCs and DLCs from deciduas according to a previously described method.^46^ This method supplies a 95% purity of vimentin^−^cytokeratin (CK)7^+^ trophoblast cells and greater than 98% vimentin^+^CK7^−^ DSCs and CD45^+^ DLCs, which was confirmed by FCM analysis.

### Enzyme-linked immunosorbent assay (ELISA)

Cytokine concentrations were measured using ELISA kits (R&D Systems).

### Detection of RANK expression on peripheral blood mononuclear cells (PBMCs) and DLCs

The PBMCs were isolated from the peripheral blood of pregnant women who were terminated for nonmedical reasons. Next, FCM was performed to analyze the expression of RANK on pMo and dM*φ* by labeling anti-human CD14, RANK and CD45 antibodies. In addition, we further evaluated the phenotype of RANK^+^ and RANK^−^ pMos and dM*φ* purified from PBMC and DLC (*n*=24) by FCM.

### Purification of dM*φ* and decidual naive T cells

We isolated dM*φ* and decidual naive T cells from DLCs by MACS (Miltenyi Biotec, Bergisch Gladbach, Germany) and performed FCM analysis with standard protocols.

### RANKL-overexpressed JEG-3 cells and DSCs

We obtained RANKL-overexpressed JEG-3 cells and DSCs by transfection with pcDNA(+)-RANKL plasmid, and the results were confirmed by FCM analysis. The pcDNA(+)-RANKL plasmid and pcDNA(+)-vector plasmid were from GeneChem Co., Ltd (Shanghai, China).

### Co-culture of trophoblasts/JEG-3 cells, DSCs and dM*φ*

The dM*φ* were cultured in culture medium, directly contacted with primary trophoblasts/JEG-3 cells and or DSCs at a 1 : 1 : 1 ratio. In addition, 5 *μ*g/ml anti-human RANKL neutralizing antibody (AB626, R&D Systems), 100 ng/ml rhOPG protein (185-OS-025, R&D Systems), 10 *μ*M LY294002 (Cells Signal Technology, Danvers, MA, USA) or 21 nM STAT6 signal inhibitor (STAT6i) AS1517499 (Axon Medchem, Groningen, The Netherlands) was added to the co-culture unit. After 48 h, the expression of RANKL on trophoblasts and DSCs, the expression of RANK, and the M1 phenotype and M2 phenotype on dM*φ* were analyzed by FCM, and the concentration of IL-10, IL-12p40 and IL-23 in the supernatants was detected by ELISA (R&D Systems).

### The intracellular phosphorylation level of Akt and STAT6

The intracellular phosphorylation level of Akt and STAT6 in dM*φ* after 24-h culture with JEG-3 and DSCs was analyzed using BD Phosflow antibodies, according to standard protocols.

### The transcription of *Jmjd3, IRF4* and *IRF5*

After 24-h co-culture, the transcription level of *Jmjd3, IRF4* and *IRF5* in dM*φ* was analyzed by real-time PCR, according to standard protocols. The primer sequences were designed and synthesized by TaKaRa Biotechnology Co., Ltd (Tokyo, Japan) as described in Supplementary Table 1.

### Co-culture of dM*φ* and decidual naive T cells

After 48 h of culture with trophoblasts/JEG-3 cells and DSCs, CD14^+^ dM*φ* were collected and washed three times with phosphate-buffered saline to remove excess cytokines. The remaining cells were co-cultured with autologous decidual naive T cells at a 1 : 1 ratio. The decidual naive T cells were pre-activated with 5 *μ*g/ml anti-CD3 (OKT3; 16-0037; ebioscience), 1 *μ*g/ml anti-CD28 (CD28.2; 16-0289; ebioscience), and 20 U/ml rhIL-2 (202-IL-010, R&D Systems) for 2 days, and collected, washed, and then incubated with culture medium only. After 5 days of co-culture, the naive T cells were reactivated with anti-CD3 and anti-CD28 for 24 h before the supernatants were collected. The expression of GATA-3 and T-bet in decidual naive T cells, and the secretion level of IL-4, IL-10, and TNF-*α* and IFN-*γ* were analyzed by FCM and ELISA (R&D Systems), respectively.

### Animals and experimental design

We divided female C57BL/6 mice (age: 8 weeks old, weight: 20–23 g) into two groups by using a random number table by body weight, age and family: the adoptive transfer of RANK^+^ M*φ* group and the adoptive transfer of RANK^−^ M*φ* group. This was an unblinded trial.

The differentiation of uM*φ* and uCD4^+^T cells, the activation of Akt and STAT6, and the level of IRF4 in uM*φ* were analyzed by FCM, and the level of fetal loss was counted at day 14 of gestation in WT and RANKL^−/−^ pregnant mice. The day of appearance of a copulatory plug was arbitrarily designated as day 0 of gestation. The embryo absorption rate and implantation number were counted on day 14 of gestation. The percentage of fetal loss (the embryo absorption rate) was calculated as follows: percentage fetal loss=R/(R+V) × 100, where R represents the number of hemorrhagic implantatio (sites of fetal loss) and V stands for the number of viable, surviving fetuses.

In addition, mouse uterine tissues were removed, minced on ice and digested with an enzyme mix of Liberase and Dispase (Invitrogen, Carlsbad, CA, USA). uM*φ* were isolated from mouse uterus by MACS (Miltenyi Biotec) to analyze the transcription of *Jmid3* and *IRF4* (Takara Bio Inc.,Tokyo, Japan) by RT-PCR at day 10 of gestation. The primer sequences were described in Supplementary Table 1.

For M*φ* depletion and M*φ* adoptive transfer in pregnant C57BL/6 mice, and Clodronate Liposomes were injected intraperitoneally at day 1 (200 *μ*l) and day 4 (100 *μ*l) of gestation, and then the expression of RANKL in Vimentin^+^ uSC and CK7^+^ pTros were analyzed at day 7 by FCM. RANK^+^ and RANK^−^ M*φ*s were isolated from mouse spleen and labeled with PKH-67, and then they were transferred to M*φ*-depleted pregnant mice at day 5 of gestation. Subsequently, the differentiation of uM*φ* and uCD4^+^T cells, the activation of Akt and STAT6, and the IRF4 level in uM*φ* were analyzed by FCM at day 10 of gestation, and the level of fetal loss was counted at day 14 of gestation in the RANK^+^ transfer group and RANK^−^ transfer group.

To investigate the role of STAT6 and Jmjd3 signals in uM*φ* and uCD4^+^T cells, the pregnant C57BL/6 mice were intraperitoneally injected with STAT6i AS1517499 (200 *μ*l at the concentration of 2 mg/kg) or JMJD3i GSK J4 HCl (200 *μ*l at a concentration of 4.48 mg/kg) in (*n*=5 mice per group) at day 4, and then the differentiation of uM*φ* and uCD4^+^T cells and the IRF4 level in uM*φ* cells at day 10 were determined by FCM.

### Statistics

The continuous variable is shown as the mean±S.E.M. Continuous variables were analyzed by Student’s *t*-test in case of two groups and by one-way ANOVA using Tukey’s post-hoc test in multiple groups. The embryo resorbing rate was analyzed using an adjusted *t*-test. All analyses were conducted with SPSS 16.0 Statistical Package for the Social Sciences software (IBM SPSS, Armonk, NY, USA). *P*<0.05 was considered statistically significant.

## Publisher’s Note

Springer Nature remains neutral with regard to jurisdictional claims in published maps and institutional affiliations.
