# Supplementary material for: RANKL-mediated harmonious dialogue between fetus and mother guarantees smooth gestation by inducing decidual M2 macrophage polarization
Source: Cell Death Dis. 2017 Oct 12;8(10):e3105–. doi: 10.1038/cddis.2017.505 (PMC5682671; doi:10.1038/cddis.2017.505)
Supplement: Supplementary Table 1 [file cddis2017505x1.doc]

**Supplementary Table 1. Sequences of primers used in the paper**

**Human IRF4 sense primer： 5’-TGCTCAACAAAACAGTGGACAT-3’**

**Human IRF4 antisense primer: 5’-TGA ATAGAGGAATGGCGGATAG-3’**

**Human Jmjd3 sense primer： 5’-AGTGAGGATGAGGAGTCAGAGG-3’**

**Human Jmjd3 antisense primer: 5’-TGA TGATGTGATGGTTCTTCG-3’**

**Human IRF5 sense primer： 5’-CAAGGAGACAGG GAAATACACC-3’**

**Human IRF5 antisense primer: 5’-CATTGGAGCAGACCTCGTAGAT-3’**

**Human GAPDH sense primer： 5’-AGAAGGCTGGGG CTCATTTG-3’**

**Human GAPDH antisense primer: 5’-AGGGGCCATCCACAGTCTTC-3’**

**Mouse IRF4 sense primer: 5’-CGTCGTCTGCATTAGAAGATC-3’**

**Mouse IRF4 antisense primer: 5’-GTGTAATCCCATCTTCTATTG-3’**

**Mouse Jmjd3 sense primer: 5’-AGCAAGTCTGGAATTTGCTGC-3’**

**Mouse Jmjd3 antisense primer: 5’-GACACAGCCATGTAGGGATTC-3’**

**Mouse GAPDH sense primer: 5’-GTCTACTGGTGTCTTCACCA-3’**

**Mouse GAPDH antisense primer: 5’-GTGGCAGTGATGGCATGGAC-3’**
