# Supplementary material for: RANKL-mediated harmonious dialogue between fetus and mother guarantees smooth gestation by inducing decidual M2 macrophage polarization
Source: Cell Death Dis. 2017 Oct 12;8(10):e3105–. doi: 10.1038/cddis.2017.505 (PMC5682671; doi:10.1038/cddis.2017.505)
Supplement: Supplementary Information [file cddis2017505x2.doc]

**Supplementary Information**

**Mice**

Inbred strains of female 8-wk-old CBA/J (H-2k) and male DBA/2 (H-2d) and BALB/c (H-2d) mice were obtained from the Jackson Laboratories, and subsequently were maintained in the Laboratory Animal Facility of Fudan University (Shanghai, China). A group of adult female C57BL/6 mice were purchased from the Laboratory Animal Facility of Fudan University and used for this study. They were usually maintained for 2 wk in the animal facility before use. The Animal Care and Use Committee of Obstetrics and Gynecology Hospital, Fudan University approved all animal protocols.

**Antibody for FCM analysis.**

DLCs were stained with APC anti-human CD45 antibody (304012), [FITC anti-human CD3](http://www.bio-city.net/index.php/Product/conts/id/24600) (300306), [FITC anti-human CD4](http://www.bio-city.net/index.php/Product/conts/id/24654) (300506), FITC anti-human CD14 antibody (301804), [PE anti-human CD56](http://www.bio-city.net/index.php/Product/conts/id/25268) (304605), [PE anti-human TCR γ/δ](http://www.bio-city.net/index.php/Product/conts/id/27340) (331210). pMo and dMφ were stained with FITC-conjugated anti-human CD14 antibody (301804), phycoerythrin -cyanine 7 (PE-Cy7)-conjugated CD80 antibody (305218), APC-conjugated CD86 antibody (305412), PE-conjugated HLA-DR antibody (307606), APC-conjugated CD206 (321110), allophycocyanin-cyanine 5.5 ([PerCP/Cy5.5](http://www.bio-city.net/index.php/Product/conts/id/777075))-conjugated CD209 antibody (330110), PE-conjugated CD163 antibody (333606), PE-conjugated IL-12/23p40 antibody (501807), PE-Cy7-conjugated IL-10 antibody (501420), and APC-conjugated CD11c antibody (301614) (all from Biolegend).

The uMφ or spleen Mφ was stained with [PE anti-mouse RANK antibody (119805)](http://www.bio-city.net/index.php/Product/conts/id/23424), [Alexa Fluor® 647 anti-mouse F4/80](http://www.bio-city.net/index.php/Product/conts/id/280746) antibody (122610), [PE/Cy7 anti-mouse CD45](http://www.bio-city.net/index.php/Product/conts/id/22074) antibody (103114), FITC anti-mouse CD11b antibody (101206), [FITC anti-mouse CD80](http://www.bio-city.net/index.php/Product/conts/id/22300) antibody (104706), [PE anti-mouse CD86](http://www.bio-city.net/index.php/Product/conts/id/22331) antibody (105008), [FITC anti-mouse CD206 (MMR)](http://www.bio-city.net/index.php/Product/conts/id/144646) antibody (141704), Brilliant Violet 421^TM^ (BV421) [anti-mouse IL-10](http://www.bio-city.net/index.php/Product/conts/id/144646) antibody (505021), FITC anti-mouse CCR2 antibody (150607) (all from Biolegend) and PE-anti-mouse CD209 (DC-SIGN) antibody (12-2091, ebioscience).

**Other Reagents.**

Recombinant human OPG protein, anti-human and anti-mouse RANKL neutralizing antibodies were from R&D Systems; LY294002 were purchased from Cell Signal Technology (USA); The pcDNA(+)-RANKL plasmid and pcDNA(+)-vector plasmid were from GeneChem Co., Ltd (China); Lipofectamine 2000 was from Invitrogen (USA). Human placental choriocarcinoma cell line (JEG-3 cells) was purchased from Bank of Cell, Chinese Academy of Sciences, Shanghai, China.

**Co-culture of trophoblasts cells, DSCs and dMφ.** The dMφ were cultured with culture medium, directly contacted with the primary trophoblasts and or DSCs. We also added 5 ug/ml anti-human RANKL neutralizing antibody (AB626, R&D) or 100ng/ml rhOPG protein ([185-OS-025](http://www.rndsystems.com/Products/185-OS), R&D), 10 uM LY294002 (cells signal technology) in co-culture unit. After 48h, the expression of M1 phenotype and M2 phenotype on dMφ were analyzed by FCM, the concentration of IL-10, IL-12p40 and IL-23 in the supernatants was detected by ELISA (R&D), and the transcription level of *Jmjd3, IRF4* and *IRF5* in dMφ was analyzed by real-time PCR.

**Animals and experimental design.** We divided female CBA/J mice (age: 8 weeks old, weight: 20g-23g) into two groups such as CBA/J♀×DBA/2♂ mating group and CBA/J♀×BALB/c♂, female C57BL/6 mice (age: 8 weeks old, weight: 20g-23g) into two groups such as adoptive transfer of RANK^+^ Mφ group and adoptive transfer of RANK^-^ Mφ group by using the table of random number by body weight, age and family.

The CBA/J♀×DBA/2♂ mating with very high fetal resorption rates were used as the abortion-prone model, whereas CBA/J♀×BALB/c♂ mating were used as a normal pregnancy model with low desorption rates. The day of appearance of a copulatory plug was arbitrarily designated as Day 0 of gestation. To investigate the role of RANKL in the differentiation of uterine Mφ (uMφ), RANK expression and phenotype of mice uterine Mφ at the day 5 and 9 of gestation were analyzed by FCM.

**Supplementary Figure 1: Phenotype of RANK^+^ and RANK^-^ pMo and Mφ.**

**a.** Decidual leukocyte cells (DLCs) were primarily isolated from human deciduas tissue of normal pregnant women (n=6) in the first-trimester, then the expression of RANK on decidual NK cells (CD45^+^CD3^-^CD56^+^), NKT cells (CD45^+^CD3^+^CD56^+^), CD3^+^T cells (CD45^+^CD3^+^), CD4^+^ T cells (CD45^+^CD4^+^), macrophages (CD45^+^CD14^+^), and γδT cells (CD45^+^CD3^+^γδTCR^+^) was analyzed by flow cytometry.

**b.** PMo and Mφ were purified from PBMC and DLC of normal pregnant women (n=24) by MACS, and FCM was performed to further analyze the phenotype of RANK^+^ and RANK^-^ pMo and Mφ by labeling CD14, RANK, CD163, CD206, CD209, CD80, CD86, HLA-DR, CD11c, IL-12/23p40, and IL-10. (One-way ANOVA)

pMo: monocytes of peripheral blood; Mφ: decidual macrophages; uMφ: uterus Mφ. Data are expressed as the mean±SEM. **P*<0.05, ***P*<0.01 and ****P*<0.001.

**Supplementary Figure 2: RANKL derived from trophoblasts and DSCs regulates dMφ differentiation.**

**a,b.** We co-cultured dMφ (n=5) with trophoblasts and or DSCs, and then incubated with recombinant human OPG protein (rhOPG, 100ng/ml) or anti-human RANKL neutralizing antibody (α-RANKL, 5ug/ml) for 48h, then the expression of CD80, CD86 and HLA-DR on dMφ was analyzed by FCM, and the secretion of IL-10, IL-12p40 and IL-23 in dMφ was analyzed by ELISA.

Data are expressed as the mean±SEM. **P*<0.05 and ***P*<0.01. ^#^*P*<0.05 and ^##^*P*<0.01 vs. dMφ alone (One-way ANOVA).

**Supplementary Figure 3: RANKL derived from trophoblasts and DSCs promotes the transcription of *Jmjd3* and *IRF4* in dMφ.**

dMφ were co-cultured with trophoblasts and DSC, and treated with or without rhOPG or α-RANKL for 24h. Then the transcription level of *Jmjd3* and *IRF4* in dMφ (n=5) was analyzed by FCM. Data are expressed as the mean±SEM. ***P*<0.01 and ****P*<0.001 (One-way ANOVA).

**Supplementary Figure 4: The RANK expression on uterus Mφ in non-pregnant and pregnant mice.**

The expression of RANK on uterus CD45^+^F4/80^+^Mφ in non-pregnant mice and pregnant mice (n=7 mice/group) by FCM. (Student’s *t*-test) Data are expressed as the mean±SEM. ****P*<0.001.

**Supplementary Figure 5: The identification of Mφ depletion in C57BL/6 mice.**

**a,b.** The Mφ depletion in C57BL/6 mice (n=5 mice/group) by intraperitoneal injection of Clodronate Liposomes at day 1 (200ul) and day 4 (100ul), and then the percentage of Mφ in mice liver, spleen and uterus at day 0, day 2, day 4, day 6 and day 7 were analyzed by FCM. **(**One-way ANOVA)

**c.** C57BL/6 mice (n=5 mice/group) with pessary was considered as pregnancy day 1 (D1), and intraperitoneal injected Clodronate Liposomes at day 1 (200ul) and day 4 (100ul), and then the expression of RANKL in Vimentin^+^ uterus stromal cells (uSC) and CK7^+^ placenta trophoblasts (pTros) at day 7 were analyzed by FCM. (Student’s *t*-test)输入文字或网址，即可翻译

Mφ^-/-^: Mφ depletion. Data are expressed as the mean±SEM. ****P*<0.001 compared to Day 0. NS: no statistically difference.

**Supplementary Figure 6: RANK^+^ macrophages with high CCR2 preferentially recruited to the uterus.**

**a.** RANK^+^ and RANK^-^ Mφs from mice spleen (n=7 mice/group) was isolated, and observed the phenotype of these RANK^+^ Mφ and RANK^-^ Mφby FCM.

**b.** FCM identification of PKH-67-RANK^+^Mφ and PKH-67-RANK^-^Mφ transfer in pregnant mice uterus (n=5 mice/group) at day 10.

c. The CCR2 expression on PKH-67-RANK^+^Mφ and PKH-67-RANK^-^ in pregnant mice uterus (n=5 mice/group) at day 10.

RANK^+^: adoptive transfer of PKH-67-RANK^+^Mφ; RANK^-^: adoptive transfer of PKH-67-RANK^-^Mφ. Data are expressed as the mean±SEM. ***P*<0.01 and ****P*<0.001 (Student’s *t*-test)
