# Supplementary figures and images for: RANKL-mediated harmonious dialogue between fetus and mother guarantees smooth gestation by inducing decidual M2 macrophage polarization
Source: Cell Death Dis. 2017 Oct 12;8(10):e3105–. doi: 10.1038/cddis.2017.505 (PMC5682671; doi:10.1038/cddis.2017.505)

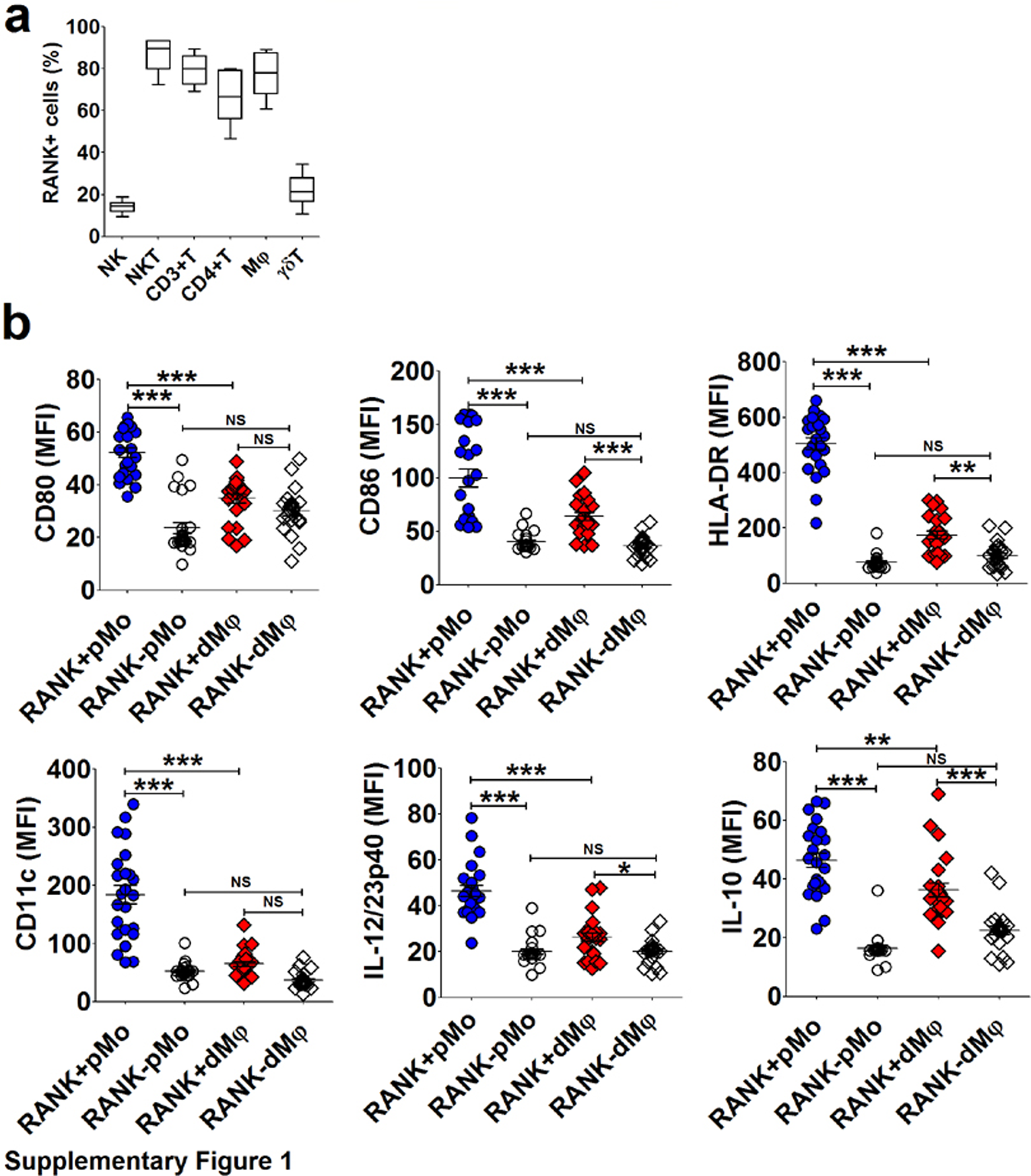

Supplement: Supplementary Figure 1 [file cddis2017505x3.tif]

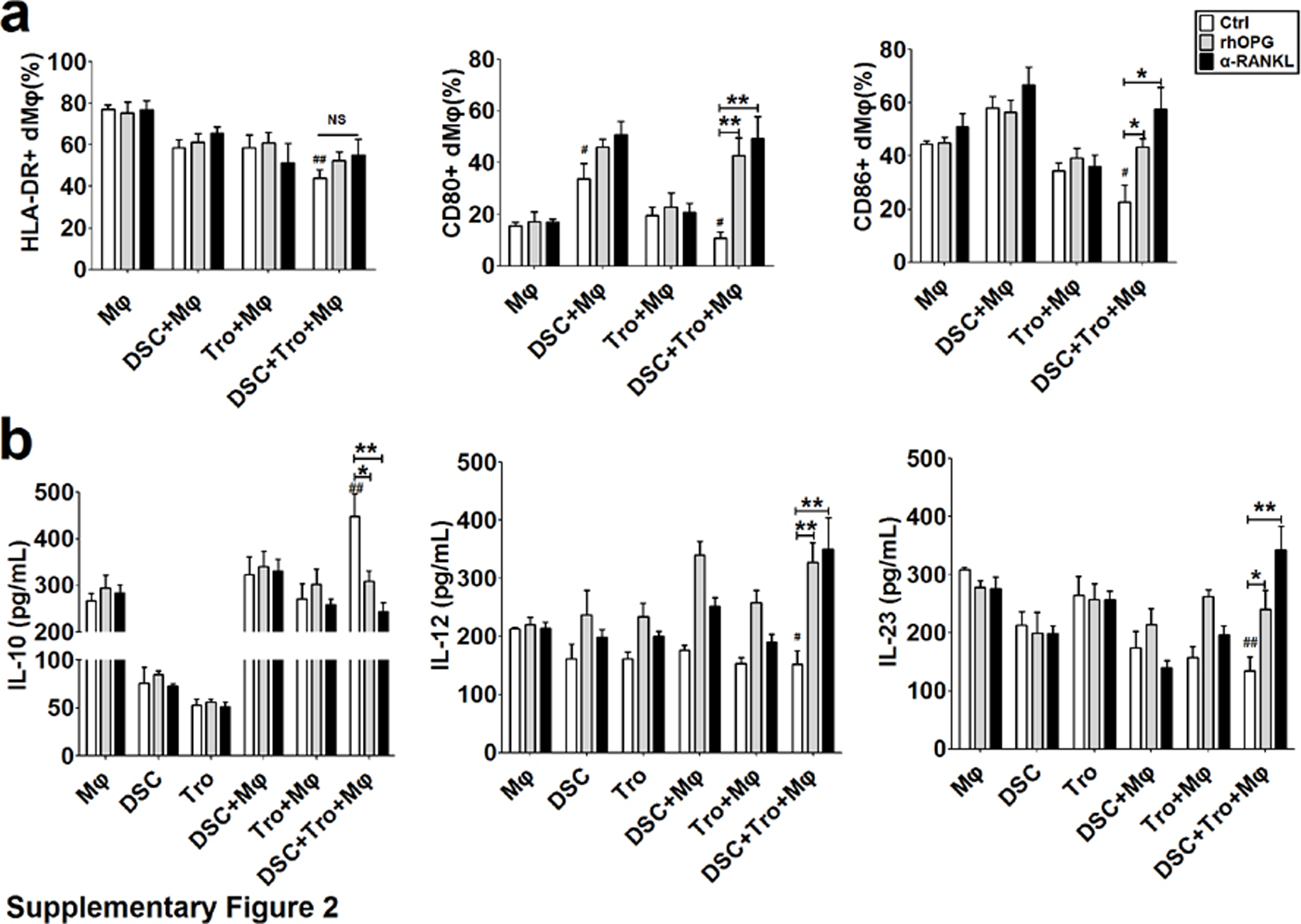

Supplement: Supplementary Figure 2 [file cddis2017505x4.tif]

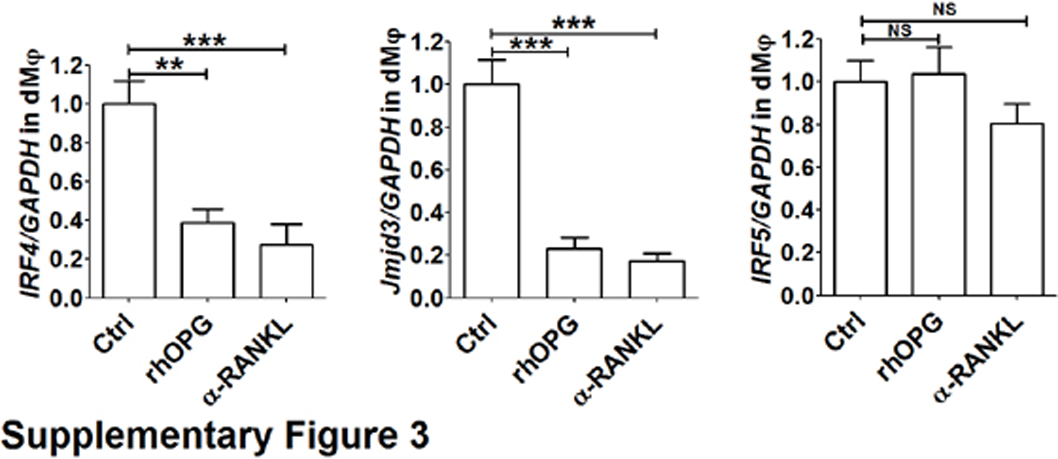

Supplement: Supplementary Figure 3 [file cddis2017505x5.tif]

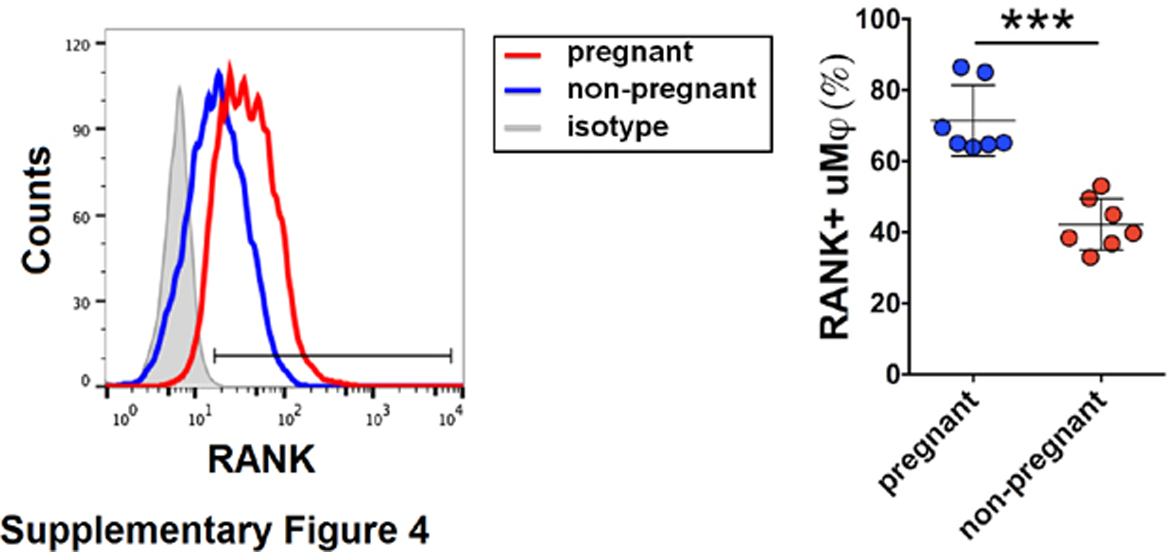

Supplement: Supplementary Figure 4 [file cddis2017505x6.tif]

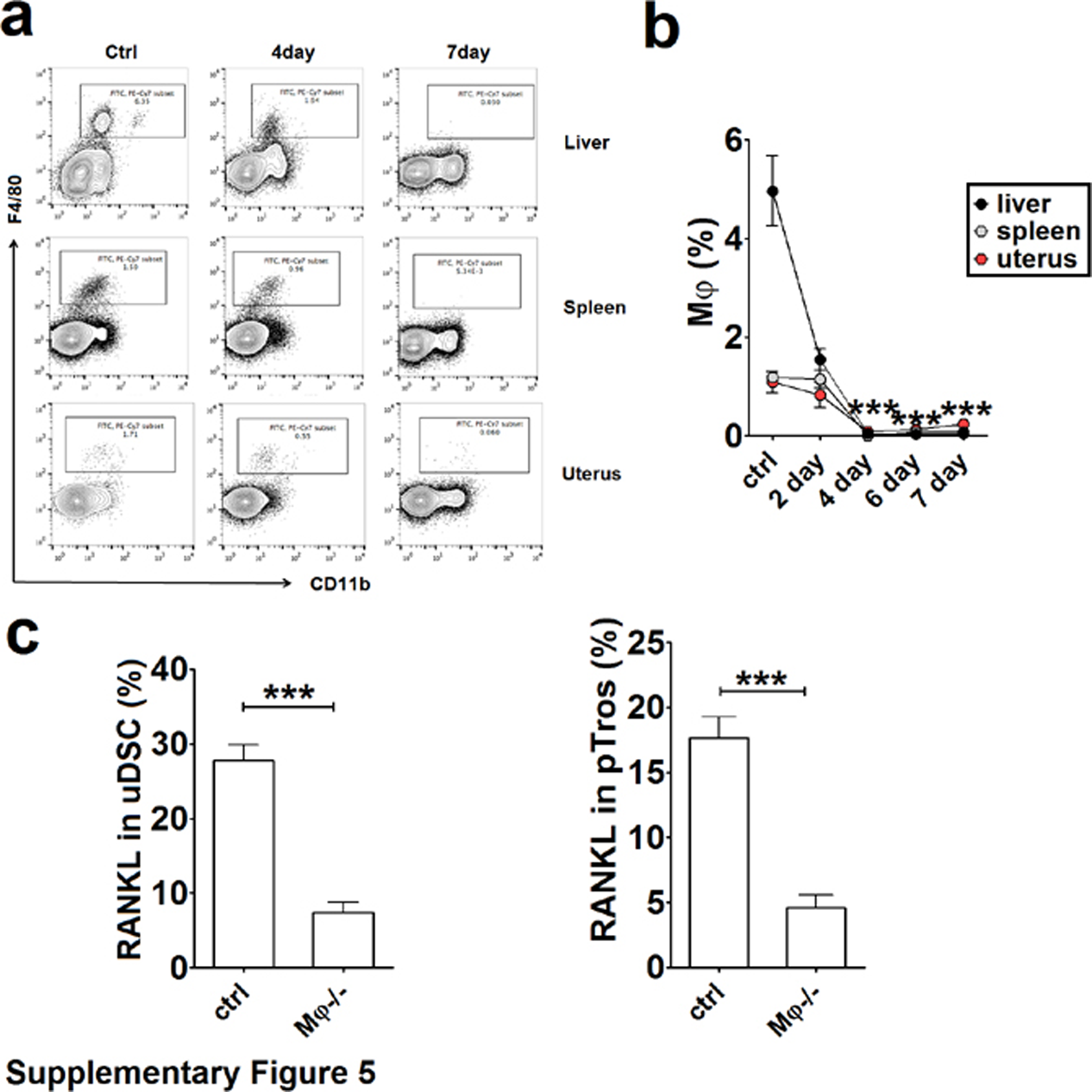

Supplement: Supplementary Figure 5 [file cddis2017505x7.tif]

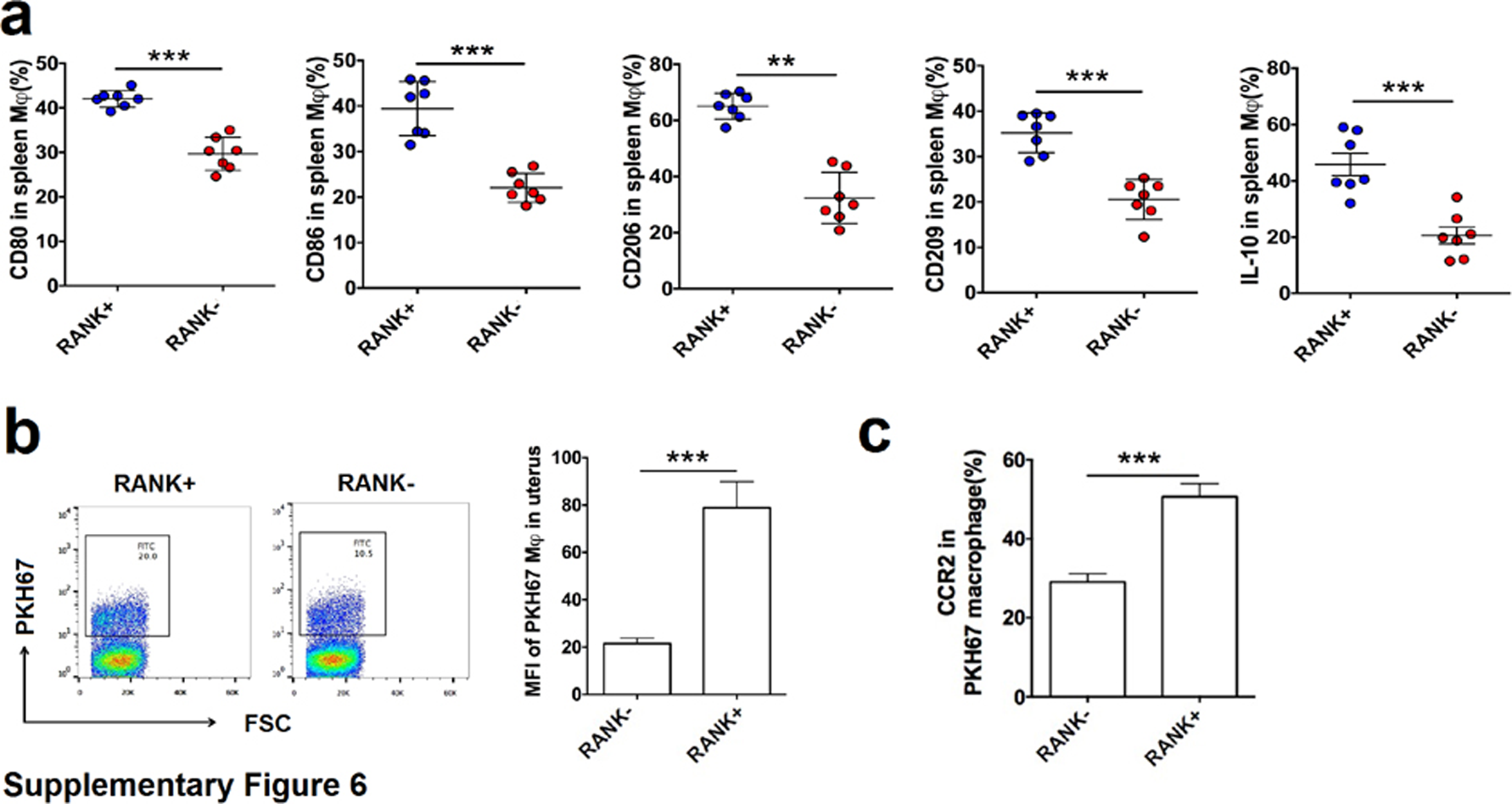

Supplement: Supplementary Figure 6 [file cddis2017505x8.tif]
